# Ethyl 2-[2-(2-meth­oxy­phen­yl)hydrazinyl­idene]-3-oxobutano­ate

**DOI:** 10.1107/S1600536811034854

**Published:** 2011-09-14

**Authors:** Hoong-Kun Fun, Madhukar Hemamalini, Shobhitha Shetty, BalaKrishna Kalluraya

**Affiliations:** a X-ray Crystallography Unit, School of Physics, Universiti Sains Malaysia, 11800 USM, Penang, Malaysia; b Department of Studies in Chemistry, Mangalore University, Mangalagangotri, Mangalore 574 199, India

## Abstract

In the title compound, C_13_H_16_N_2_O_4_, an intra­molecular N—H⋯O hydrogen bond generates an *S*(6) ring. The mol­ecule adopts an *E* configuration with respect to the central C=N double bond. In the crystal, symmetry-related mol­ecules are connected into chains along [010] *via* weak C—H⋯N hydrogen bonds. The crystal structure is further stabilized by weak C—H⋯π inter­actions.

## Related literature

For details and applications of pyrazole derivatives, see: Rai *et al.* (2008 ▶); Girisha *et al.* (2010 ▶); Isloor *et al.* (2009 ▶). For hydrogen-bond motifs, see: Bernstein *et al.* (1995 ▶). For the stability of the temperature controller used in the data collection, see: Cosier & Glazer (1986 ▶).
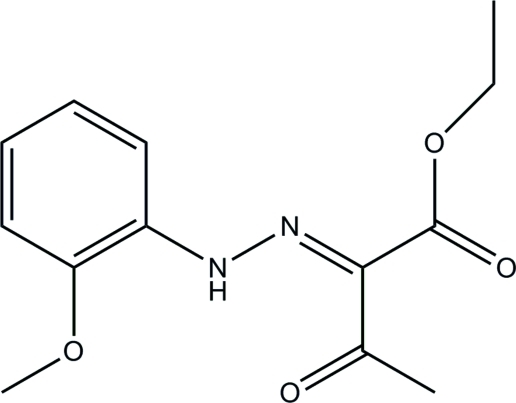


## Experimental

### 

#### Crystal data

C_13_H_16_N_2_O_4_
*M* *_r_* = 264.28
Monoclinic, 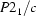

*a* = 10.1885 (4) Å
*b* = 11.4967 (4) Å
*c* = 13.2492 (5) Å
β = 120.003 (3)°
*V* = 1343.97 (9) Å^3^
*Z* = 4
Mo *K*α radiation
μ = 0.10 mm^−1^
*T* = 100 K
0.75 × 0.27 × 0.20 mm

#### Data collection

Bruker SMART APEXII CCD area-detector diffractometer
Absorption correction: multi-scan (*SADABS*; Bruker, 2009 ▶) *T*_min_ = 0.931, *T*_max_ = 0.981
14963 measured reflections
3909 independent reflections
3123 reflections with *I* > 2σ(*I*)
*R*_int_ = 0.028

#### Refinement

*R*[*F* ^2^ > 2σ(*F* ^2^)] = 0.044
*wR*(*F* ^2^) = 0.116
*S* = 1.03
3909 reflections
179 parameters
H atoms treated by a mixture of independent and constrained refinement
Δρ_max_ = 0.35 e Å^−3^
Δρ_min_ = −0.30 e Å^−3^

### 

Data collection: *APEX2* (Bruker, 2009 ▶); cell refinement: *SAINT* (Bruker, 2009 ▶); data reduction: *SAINT*; program(s) used to solve structure: *SHELXTL* (Sheldrick, 2008 ▶); program(s) used to refine structure: *SHELXTL*; molecular graphics: *SHELXTL*; software used to prepare material for publication: *SHELXTL* and *PLATON* (Spek, 2009 ▶).

## Supplementary Material

Crystal structure: contains datablock(s) global, I. DOI: 10.1107/S1600536811034854/lh5322sup1.cif

Structure factors: contains datablock(s) I. DOI: 10.1107/S1600536811034854/lh5322Isup2.hkl

Supplementary material file. DOI: 10.1107/S1600536811034854/lh5322Isup3.cml

Additional supplementary materials:  crystallographic information; 3D view; checkCIF report

## Figures and Tables

**Table 1:** Hydrogen-bond geometry (Å, °) *Cg*1 is the centroid of the C1–C6 ring.

*D*—H⋯*A*	*D*—H	H⋯*A*	*D*⋯*A*	*D*—H⋯*A*
N1—H1*N*1⋯O3	0.90 (2)	1.886 (19)	2.5715 (15)	131.6 (14)
C13—H13*C*⋯N2^i^	0.96	2.58	3.4835 (18)	156
C12—H12*B*⋯*Cg*1^ii^	0.96	2.92	3.6620 (15)	135
C13—H13*B*⋯*Cg*1^iii^	0.96	2.66	3.4887 (14)	145

Symmetry codes: (i) 
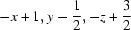
; (ii) 
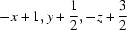
; (iii) 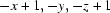.

**Fig. 1.**
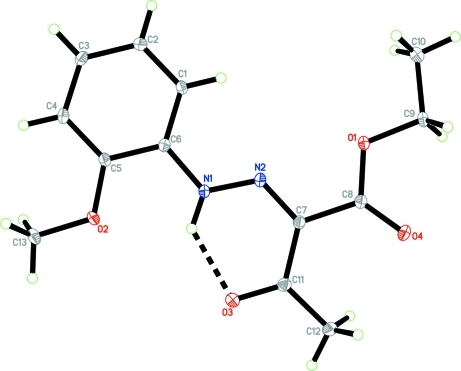
The molecular structure of the title compound, showing 50% probability displacement ellipsoids. An intramolecular hydrogen bond is shown by a dashed line.

**Fig. 2.**
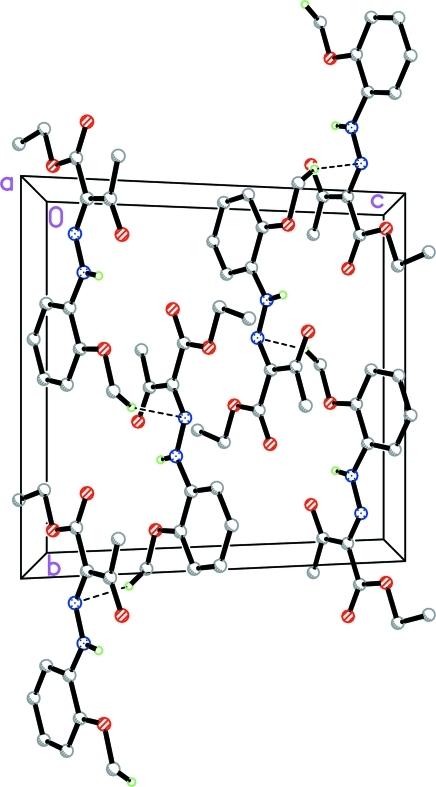
The crystal packing of the title compound (I). H atoms not involing the hydrogen bond interactions are omitted for clarity. Hydrogen bonds are shown as dashed llines.

## supplementary crystallographic information

### Comment

Pyrazole derivatives are well established in the literature as important biologically effective heterocyclic compounds (Rai *et al.*, 2008). These derivatives are the subject of many research studies due to their widespread potential pharmacological activities such as antiinflammatory (Girisha *et al.*, 2010), antipyretic, antimicrobial (Isloor *et al.*, 2009) and antiviral activities. The widely prescribed anti-inflammatory pyrazole derivatives, celecoxib and deracoxib, are selective COX-2 inhibitors with reduced ulcerogenic side effects.  The title compound, ethyl-2-[(2-methoxyphenyl) hydrazinylidene]-3-oxobutanoate is a key intermediate in the preparation of pyrazole derivative which in turn was obtained by the condensation of 2-[(2-substituted phenyl)hydrazinylidene]-3-oxobutanoate with thiosemicarbazide in glacial acetic acid medium.

Fig. 1 shows the molecular structure of the title compound (I). The molecule adopts an *E*-configuration with respect to the central C6═N1 double bond. An intramolecular N1—H1N1···O3 interaction generates an *S*(6) ring (Bernstein *et al.*, 1995). In the crystal, (Fig. 2), adjacent molecules are interconnected into one-dimensional chains along [010] *via* intermolecular C13—H13C···N2^i^ hydrogen bonds. Furthermore, the crystal structure is stabilized by C—H···π interactions (Table 1) involving the C1–C6 (centroid  Cg1) ring.

### Experimental

The title compound was prepared by dissolving 2-methoxy aniline (0.01 mol) in dilute hydrochloric acid (10 ml) and cooled to 273K in an ice bath. To this, a cold solution of sodium nitrite (0.02 mol) was added. The resulting diazonium salt  solution was filtered into a cold solution of ethyl acetoacetate (0.05 mol) and sodium acetate in ethanol. The separated yellow solid was filtered, washed with water and recrystallized from ethanol. Crystals suitable for X-ray analysis were obtained by slow evaporationfrom of a solution of (I) in a 1:2 mixture of DMF and ethanol.

### Refinement

Atom H1N1 was located in a difference Fourier map and refined freely [N–H = 0.898 (17) Å]. The remaining H atoms were positioned geometrically [C–H = 0.93–0.97 Å] and were refined using a riding model, with *U*_iso_(H) = 1.2 or 1.5 *U*_eq_(C).  A rotating group model was applied to the methyl groups.

### Crystal data

**Table:**

C_13_H_16_N_2_O_4_	*F*(000) = 560
*M**_r_* = 264.28	*D*_x_ = 1.306 Mg m^−^^3^
Monoclinic, *P*2_1_/*c*	Mo *K*α radiation, λ = 0.71073 Å
Hall symbol: -P 2ybc	Cell parameters from 7354 reflections
*a* = 10.1885 (4) Å	θ = 2.5–30.8°
*b* = 11.4967 (4) Å	µ = 0.10 mm^−^^1^
*c* = 13.2492 (5) Å	*T* = 100 K
β = 120.003 (3)°	Needle, green
*V* = 1343.97 (9) Å^3^	0.75 × 0.27 × 0.20 mm
*Z* = 4	

### Data collection

**Table:**

Bruker SMART APEXII CCD area-detector diffractometer	3909 independent reflections
Radiation source: fine-focus sealed tube	3123 reflections with *I* > 2σ(*I*)
graphite	*R*_int_ = 0.028
φ and ω scans	θ_max_ = 30.0°, θ_min_ = 2.3°
Absorption correction: multi-scan (*SADABS*; Bruker, 2009)	*h* = −14→13
*T*_min_ = 0.931, *T*_max_ = 0.981	*k* = −16→16
14963 measured reflections	*l* = −14→18

### Refinement

**Table:**

Refinement on *F*^2^	Primary atom site location: structure-invariant direct methods
Least-squares matrix: full	Secondary atom site location: difference Fourier map
*R*[*F*^2^ > 2σ(*F*^2^)] = 0.044	Hydrogen site location: inferred from neighbouring sites
*wR*(*F*^2^) = 0.116	H atoms treated by a mixture of independent and constrained refinement
*S* = 1.03	*w* = 1/[σ^2^(*F*_o_^2^) + (0.0513*P*)^2^ + 0.4458*P*] where *P* = (*F*_o_^2^ + 2*F*_c_^2^)/3
3909 reflections	(Δ/σ)_max_ < 0.001
179 parameters	Δρ_max_ = 0.35 e Å^−^^3^
0 restraints	Δρ_min_ = −0.30 e Å^−^^3^

### Special details

**Table:**

Experimental. The crystal was placed in the cold stream of an Oxford Cryosystems Cobra open-flow nitrogen cryostat (Cosier & Glazer, 1986) operating at 100.0 (1) K.
Geometry. All esds (except the esd in the dihedral angle between two l.s. planes) are estimated using the full covariance matrix. The cell esds are taken into account individually in the estimation of esds in distances, angles and torsion angles; correlations between esds in cell parameters are only used when they are defined by crystal symmetry. An approximate (isotropic) treatment of cell esds is used for estimating esds involving l.s. planes.
Refinement. Refinement of F^2^ against ALL reflections. The weighted R-factor wR and goodness of fit S are based on F^2^, conventional R-factors R are based on F, with F set to zero for negative F^2^. The threshold expression of F^2^ > 2sigma(F^2^) is used only for calculating R-factors(gt) etc. and is not relevant to the choice of reflections for refinement. R-factors based on F^2^ are statistically about twice as large as those based on F, and R- factors based on ALL data will be even larger.

### Fractional atomic coordinates and isotropic or equivalent isotropic displacement parameters (Å^2^)

**Table:**

	*x*	*y*	*z*	*U*_iso_*/*U*_eq_	
O1	0.30643 (9)	0.58032 (7)	0.53563 (7)	0.02056 (18)	
O2	0.61515 (9)	0.07478 (7)	0.68475 (7)	0.01987 (18)	
O3	0.76423 (9)	0.37484 (7)	0.73708 (7)	0.02157 (19)	
O4	0.51710 (10)	0.68753 (7)	0.63602 (7)	0.02266 (19)	
N1	0.49890 (11)	0.28280 (8)	0.62667 (8)	0.01539 (19)	
N2	0.44571 (10)	0.38868 (8)	0.60104 (8)	0.01477 (19)	
C1	0.24246 (12)	0.20271 (10)	0.51384 (9)	0.0164 (2)	
H1A	0.2010	0.2769	0.4937	0.020*	
C2	0.14890 (13)	0.10570 (10)	0.47404 (10)	0.0188 (2)	
H2A	0.0447	0.1147	0.4267	0.023*	
C3	0.21166 (13)	−0.00471 (10)	0.50522 (10)	0.0193 (2)	
H3A	0.1486	−0.0695	0.4788	0.023*	
C4	0.36743 (13)	−0.02020 (10)	0.57532 (10)	0.0182 (2)	
H4A	0.4082	−0.0946	0.5955	0.022*	
C5	0.46119 (12)	0.07678 (9)	0.61481 (9)	0.0155 (2)	
C6	0.39815 (12)	0.18864 (9)	0.58381 (9)	0.0149 (2)	
C7	0.53715 (12)	0.47955 (9)	0.63721 (9)	0.0154 (2)	
C8	0.45682 (13)	0.59306 (9)	0.60453 (9)	0.0160 (2)	
C9	0.21993 (13)	0.68756 (10)	0.50635 (10)	0.0211 (2)	
H9A	0.2528	0.7404	0.4663	0.025*	
H9B	0.2336	0.7254	0.5764	0.025*	
C10	0.05623 (14)	0.65495 (11)	0.42841 (12)	0.0297 (3)	
H10A	−0.0054	0.7237	0.4075	0.045*	
H10B	0.0255	0.6022	0.4689	0.045*	
H10C	0.0442	0.6182	0.3592	0.045*	
C11	0.70375 (12)	0.47143 (10)	0.70367 (9)	0.0172 (2)	
C12	0.80061 (13)	0.57743 (11)	0.72907 (11)	0.0232 (2)	
H12A	0.9049	0.5546	0.7633	0.035*	
H12B	0.7885	0.6267	0.7823	0.035*	
H12C	0.7706	0.6189	0.6579	0.035*	
C13	0.68844 (14)	−0.03669 (10)	0.71190 (10)	0.0215 (2)	
H13A	0.7964	−0.0261	0.7543	0.032*	
H13B	0.6599	−0.0782	0.6410	0.032*	
H13C	0.6581	−0.0803	0.7586	0.032*	
H1N1	0.5992 (19)	0.2707 (14)	0.6701 (14)	0.037 (4)*	

### Atomic displacement parameters (Å^2^)

**Table:**

	*U*^11^	*U*^22^	*U*^33^	*U*^12^	*U*^13^	*U*^23^
O1	0.0175 (4)	0.0120 (4)	0.0265 (4)	0.0022 (3)	0.0067 (3)	−0.0010 (3)
O2	0.0152 (4)	0.0145 (4)	0.0242 (4)	0.0039 (3)	0.0056 (3)	0.0015 (3)
O3	0.0179 (4)	0.0180 (4)	0.0241 (4)	0.0013 (3)	0.0069 (3)	0.0013 (3)
O4	0.0229 (4)	0.0128 (4)	0.0280 (4)	−0.0026 (3)	0.0095 (4)	−0.0013 (3)
N1	0.0153 (4)	0.0113 (4)	0.0177 (4)	0.0009 (4)	0.0068 (4)	0.0009 (3)
N2	0.0182 (4)	0.0114 (4)	0.0149 (4)	0.0006 (3)	0.0084 (4)	0.0001 (3)
C1	0.0168 (5)	0.0132 (5)	0.0191 (5)	0.0030 (4)	0.0088 (4)	0.0021 (4)
C2	0.0160 (5)	0.0173 (5)	0.0224 (5)	−0.0001 (4)	0.0092 (4)	−0.0001 (4)
C3	0.0202 (5)	0.0144 (5)	0.0233 (5)	−0.0034 (4)	0.0108 (5)	−0.0019 (4)
C4	0.0224 (6)	0.0116 (5)	0.0208 (5)	0.0010 (4)	0.0111 (4)	0.0006 (4)
C5	0.0163 (5)	0.0139 (5)	0.0158 (5)	0.0021 (4)	0.0077 (4)	0.0006 (4)
C6	0.0173 (5)	0.0122 (5)	0.0163 (5)	−0.0005 (4)	0.0092 (4)	−0.0002 (4)
C7	0.0168 (5)	0.0133 (5)	0.0150 (5)	−0.0002 (4)	0.0071 (4)	0.0002 (4)
C8	0.0182 (5)	0.0140 (5)	0.0158 (5)	−0.0001 (4)	0.0085 (4)	0.0001 (4)
C9	0.0210 (6)	0.0122 (5)	0.0272 (6)	0.0038 (4)	0.0100 (5)	−0.0004 (4)
C10	0.0205 (6)	0.0212 (6)	0.0420 (7)	0.0040 (5)	0.0116 (6)	0.0000 (5)
C11	0.0170 (5)	0.0177 (5)	0.0152 (5)	−0.0010 (4)	0.0067 (4)	−0.0007 (4)
C12	0.0184 (5)	0.0204 (6)	0.0264 (6)	−0.0038 (5)	0.0078 (5)	0.0001 (5)
C13	0.0215 (6)	0.0192 (6)	0.0243 (6)	0.0081 (5)	0.0119 (5)	0.0052 (4)

### Geometric parameters (Å, °)

**Table:**

O1—C8	1.3430 (14)	C4—H4A	0.9300
O1—C9	1.4510 (13)	C5—C6	1.4041 (15)
O2—C5	1.3660 (13)	C7—C11	1.4732 (16)
O2—C13	1.4355 (13)	C7—C8	1.4852 (15)
O3—C11	1.2393 (13)	C9—C10	1.5053 (17)
O4—C8	1.2143 (13)	C9—H9A	0.9700
N1—N2	1.3064 (12)	C9—H9B	0.9700
N1—C6	1.4019 (14)	C10—H10A	0.9600
N1—H1N1	0.898 (17)	C10—H10B	0.9600
N2—C7	1.3201 (14)	C10—H10C	0.9600
C1—C2	1.3885 (15)	C11—C12	1.4967 (16)
C1—C6	1.3896 (15)	C12—H12A	0.9600
C1—H1A	0.9300	C12—H12B	0.9600
C2—C3	1.3880 (16)	C12—H12C	0.9600
C2—H2A	0.9300	C13—H13A	0.9600
C3—C4	1.3925 (16)	C13—H13B	0.9600
C3—H3A	0.9300	C13—H13C	0.9600
C4—C5	1.3891 (15)		
			
C8—O1—C9	115.03 (9)	O1—C8—C7	112.17 (9)
C5—O2—C13	117.52 (9)	O1—C9—C10	106.76 (9)
N2—N1—C6	119.32 (9)	O1—C9—H9A	110.4
N2—N1—H1N1	120.1 (11)	C10—C9—H9A	110.4
C6—N1—H1N1	120.6 (11)	O1—C9—H9B	110.4
N1—N2—C7	121.15 (9)	C10—C9—H9B	110.4
C2—C1—C6	119.85 (10)	H9A—C9—H9B	108.6
C2—C1—H1A	120.1	C9—C10—H10A	109.5
C6—C1—H1A	120.1	C9—C10—H10B	109.5
C3—C2—C1	119.66 (10)	H10A—C10—H10B	109.5
C3—C2—H2A	120.2	C9—C10—H10C	109.5
C1—C2—H2A	120.2	H10A—C10—H10C	109.5
C2—C3—C4	121.15 (10)	H10B—C10—H10C	109.5
C2—C3—H3A	119.4	O3—C11—C7	119.26 (10)
C4—C3—H3A	119.4	O3—C11—C12	119.68 (10)
C5—C4—C3	119.23 (10)	C7—C11—C12	121.05 (10)
C5—C4—H4A	120.4	C11—C12—H12A	109.5
C3—C4—H4A	120.4	C11—C12—H12B	109.5
O2—C5—C4	125.60 (10)	H12A—C12—H12B	109.5
O2—C5—C6	114.58 (9)	C11—C12—H12C	109.5
C4—C5—C6	119.81 (10)	H12A—C12—H12C	109.5
C1—C6—N1	122.73 (10)	H12B—C12—H12C	109.5
C1—C6—C5	120.29 (10)	O2—C13—H13A	109.5
N1—C6—C5	116.98 (9)	O2—C13—H13B	109.5
N2—C7—C11	124.05 (10)	H13A—C13—H13B	109.5
N2—C7—C8	113.80 (9)	O2—C13—H13C	109.5
C11—C7—C8	122.15 (10)	H13A—C13—H13C	109.5
O4—C8—O1	122.70 (10)	H13B—C13—H13C	109.5
O4—C8—C7	125.13 (10)		
			
C6—N1—N2—C7	−178.24 (9)	C4—C5—C6—N1	179.90 (10)
C6—C1—C2—C3	0.46 (16)	N1—N2—C7—C11	2.76 (16)
C1—C2—C3—C4	−0.43 (17)	N1—N2—C7—C8	−177.39 (9)
C2—C3—C4—C5	0.18 (17)	C9—O1—C8—O4	−3.10 (15)
C13—O2—C5—C4	5.71 (15)	C9—O1—C8—C7	176.32 (9)
C13—O2—C5—C6	−174.96 (9)	N2—C7—C8—O4	174.02 (10)
C3—C4—C5—O2	179.33 (10)	C11—C7—C8—O4	−6.12 (17)
C3—C4—C5—C6	0.03 (16)	N2—C7—C8—O1	−5.37 (13)
C2—C1—C6—N1	179.86 (10)	C11—C7—C8—O1	174.48 (9)
C2—C1—C6—C5	−0.26 (15)	C8—O1—C9—C10	179.35 (10)
N2—N1—C6—C1	0.42 (15)	N2—C7—C11—O3	−6.31 (16)
N2—N1—C6—C5	−179.46 (9)	C8—C7—C11—O3	173.85 (10)
O2—C5—C6—C1	−179.36 (9)	N2—C7—C11—C12	172.36 (10)
C4—C5—C6—C1	0.01 (15)	C8—C7—C11—C12	−7.48 (15)
O2—C5—C6—N1	0.53 (13)		

### Hydrogen-bond geometry (Å, °)

**Table:**

Cg1 is the centroid of the C1–C6 ring.

**Table:**

*D*—H···*A*	*D*—H	H···*A*	*D*···*A*	*D*—H···*A*
N1—H1N1···O3	0.90 (2)	1.886 (19)	2.5715 (15)	131.6 (14)
C13—H13C···N2^i^	0.96	2.58	3.4835 (18)	156
C12—H12B···Cg1^ii^	0.96	2.92	3.6620 (15)	135
C13—H13B···Cg1^iii^	0.96	2.66	3.4887 (14)	145

Symmetry codes: (i) −*x*+1, *y*−1/2, −*z*+3/2; (ii) −*x*+1, *y*+1/2, −*z*+3/2; (iii) −*x*+1, −*y*, −*z*+1.
